# Loneliness in young adulthood: Its intersecting forms and its association with psychological well-being and family characteristics in Northern Taiwan

**DOI:** 10.1371/journal.pone.0217777

**Published:** 2019-05-31

**Authors:** Chi Chiao, Yu-Hua Chen, Chin-Chun Yi

**Affiliations:** 1 Institute of Health and Welfare Policy, Institute of Public Health, School of Medicine, National Yang-Ming University, Taipei Taiwan; 2 Department of Bio-Industry Communication and Development, National Taiwan University, Taipei Taiwan; 3 Institute of Sociology, Academia Sinica, Taipei Taiwan; University of Sao Paulo Medical School, BRAZIL

## Abstract

**Objectives:**

Most researchers have examined forms of loneliness as discrete and emotional distress. The approach proposed in this study captures the reality that many persons experience more than one dimension of loneliness—varying degrees coupled with their psychological well-being in a family context. This study explores the latent structure of loneliness during young adulthood and its association with psychological well-being, as well as how these are related to their family characteristics in adolescence.

**Methods:**

Data are from 2,748 young people, a cohort sample from the Taiwan Youth Project (TYP). Loneliness was assessed by a 6-item de Jong-Gierveld short scale with emotional and social loneliness domains. We describe the clustering between loneliness domains and psychological well-being, namely depressive symptoms, self-esteem, suicidal thoughts, and alcohol use using latent class cluster analysis. In addition to incorporating the Taiwanese family context, multivariate multinomial logistic regression models included data on family cohesion and parental *guan* (parental control) in adolescence. This might be associated with choices in partnership and childbearing, and influence loneliness in young adulthood.

**Results:**

Our results demonstrate a three-cluster model of loneliness involving emotional loners, serious emotional loners, and severe emotional/social loners. We also found that a feeling of serious emotional loneliness and severe emotional/social loneliness were significantly associated with psychological well-being, even adjusting for individual characteristics. Among young adults who had a partner, the married adults were significantly less likely to feel serious emotional loneliness than those who were living alone. Furthermore, young adults with stronger family cohesion during early adolescence were less likely to suffer from serious emotional loneliness (Relative risk ratios [RRR] 0.77, 95% CI 0.65–0.91) and severe emotional/social loneliness (RRR 0.54, 95% CI 0.34–0.85) in young adulthood.

**Discussion:**

This measurement strategy provides a foundation for future research into how experts can address loneliness clusters in order to better understand psychological well-being during young adulthood and family context in adolescence. This is important because our results suggest that the various loneliness domains do not occur independently, but rather are embedded in patterns and are associated with family characteristics.

## Introduction

Despite international support aimed at improving mental health that has been introduced over the past few decades, loneliness remains a concern [1], particularly when it affects young adults in general [2], and those living in non-Western countries in particular [3,4]. McWhirter (1990) defines loneliness as *an enduring condition of emotional distress that arises when a person feels estranged from misunderstood or rejected by others*, *and/or lacks appropriate social partners for desired activities*, *particularly for activities that provide a sense of social integration and opportunities for emotional intimacy* [5]. Some of the recent interest in loneliness has targeted the emotional domain, and much of this analysis has not been linked to social relationships.

Scholars tend to operationalize loneliness as a set of discrete phenomena. They have also examined how the emotional domain is related to the social domain, though this approach is less common [6]. Given that many individuals experience more than one domain throughout the life course, it is plausible that the emotional and social domains may consolidate in distinct patterns [7]. To be specific, some indicators of emotional and social loneliness are complementary, while others are in competition with one another. Thus, it is reasonable to anticipate that various dimensions of loneliness may cluster [2]. To our knowledge, there have been no previous attempt to understand how this may occur and whether the multiple indicators of loneliness may be interrelated. Such relationships are germane to the goal of determining whether there is a latent structure that captures how the dimensions of loneliness [2] and psychological well-being interrelate [2–4]. If such clustering is identified, the results will be of conceptual interest. They will also help researchers understand loneliness, from both the psychological and social perspectives [2]. Prior research has found that loneliness is associated with numerous mental health problems [8,9]. Exploring latent clusters of loneliness will allow researchers to explicate how loneliness is related to psychological well-being in young adults.

Furthermore, a parallel but unconnected stream of research has explored the relationship between loneliness and family characteristics. This *risky families model* [10] has suggested that family conflict experienced early in life may affect mental health development. By extending this idea, we hypothesize that family context during adolescence can shape subsequent loneliness. However, most of these earlier studies have focused on emotional loneliness. They have not examined the consequences of various dimensional measures such as loneliness clusters. Moreover, such relationships have not been explored in detail in non-Western societies. Early detection of family environments associated with specific dimensional measures can be crucial to preventing mental health problems among young adults [2]. Exploring family influences, such as family cohesion, parental *guan* (control), partnership and childbearing choices, are further warranted to understand the relationships between the dimensional measures of loneliness, independent of other psychological well-being, and how these are linked to family contexts.

The present study assesses whether specific indicators are likely to cluster in certain loneliness domains, and if this occurs, whether these clusters have any conceptual meaning. We are especially interested in whether the indicators are complementary or in competition with one another, and whether they are emotional or social. Furthermore, how is this latent structure associated with other psychological well-being indicators, namely depressive symptoms, self-esteem, suicidal thoughts, and alcohol consumption. The present study further builds upon the framework of the risky families model [10]. It explores family cohesion and parental *guan* during early adolescence, and their association with loneliness clustering in young people, when considering their current partnership and childbearing choices.

## Methods

### Data

The dataset was extracted from the panel surveys of the Taiwan Youth Project (TYP). The TYP surveys began in 2000 when participants were in junior high school, with follow-ups in 2001, 2002, 2003, 2005, 2006, 2007, 2009, 2011, and 2014. A multi-stage random sampling frame was used to obtain school-based representative samples of junior high students in Northern Taiwan, including Taipei City, New Taipei City, and Yi-Lan County. Levels of urbanization were adopted at the first stage of sampling. This divided Taipei City and Taipei County into three strata, and Yi-Lan County into two strata. Further details of the sampling design and data collection procedures have been described in previous studies [11].

**Fig 1:**
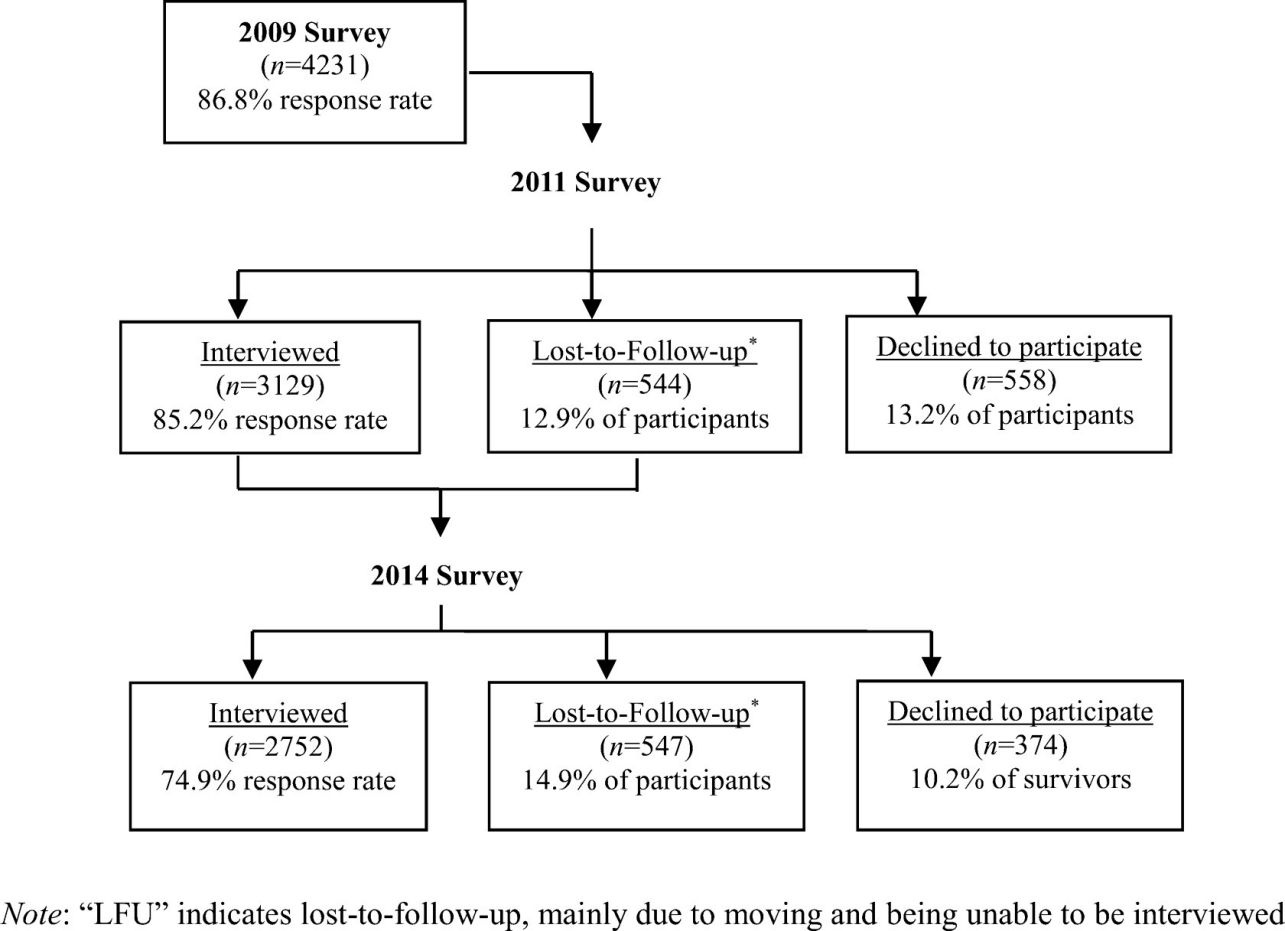
Participants in serial surveys in the Taiwan Youth Project (TYP) from 2009 to 2014.

The TYP surveys provide longitudinal information on a range of demographic and family information gathered from early adolescence to young adulthood. The first adult survey was conducted in 2011, however the TYP did not collect data on loneliness in young people until 2014, as it was around this time that participants were approaching marriage age. The response rate for the 2014 survey was 74.9%. The attrition rate was 10.2%, as compared with the 2011 survey. The adult sample flow is shown in Fig 1.

We restricted our analyses to respondents who had given complete responses for the major measures collected in 2014. This excluded 4 participants. The final sample consisted of 1,284 females and 1,464 males. TYP data are publicly available, and can be used for research with the approval of Academia Sinica in Taiwan (http://www.typ.sinica.edu.tw). All TYP participants gave informed written consent at the start of their interviews. The study protocol was approved by the Research Ethics Committee of National Yang-Ming University (Taipei, Taiwan) (IRB Number: YM106103E-1).

### Measures

#### Outcome measure

Loneliness was assessed by self-reporting via a 6-item de Jong-Gierveld short scale [12,13] which included distinct dimensions of emotional and social loneliness [14]. Each item was recoded into a dichotomous score, indicating whether the individual was not lonely (coded as 0) or was extremely lonely (coded as 1) [15]. The 6-item de Jong-Gierveld scale with its descriptive statistics and psychometrics properties are shown in S1 Appendix. The most common loneliness reported in the TYP sample of young adults was a general sense of emptiness (52%), followed by missing having people around (25%). The least common indicators were related to social loneliness. Confirmatory factor analyses on the 6 items revealed two factors with good reliability: emotional and social domains used in the TYP. The use of this 6-item de Jong-Gierveld short scale as loneliness measures has also been validated in Chinese, but with elderly people [16].

*Psychological well-being* was measured by four self-reported indicators: depressive symptoms, self-esteem, suicidal thoughts, and alcohol use. Depressive symptoms were assessed using an 8-item version of the Symptom Checklist-90 Revised (SCL-90-R) scale [17–19]. The SCL-90-R is an instrument with good construct validity and reliability when used with Taiwanese adolescents [19,20]. Each five-point scale indicates the degree to which each symptom had been experienced within the past week. When necessary, responses were reverse-scored so that higher scores indicated greater depressive symptomatology. Scores ranged from 8 to 40, with Cronbach’s alphas across waves ranging from 0.82–0.85. Self-esteem was assessed using 6 items of the Rosenberg’s Self-Esteem Scale [21]. Each item was scored from 1 (strongly agree) to 4 (strongly disagree), and scores ranged from 6 to 24 with higher scores representing higher self-esteem. Confirmatory factor analyses yielded good fit for the models (RMSEA = 0.04; CFI = 0.99) with good reliability (Cronbach’s α = 0.67). Suicidal thoughts are considered to occur across a continuum of clinical significance [22] and are an important marker of mental health problems [23]. Suicidal thoughts were measured by the question, “During the past 12 months, did you ever seriously think about committing suicide?” (1 = yes, 0 = no) [24]. Alcohol use was measured by asking the age of first alcohol consumption (either adolescence, adulthood, and complete abstainer) and whether the subject was a binge drinker (1 = yes, 0 = no) [25].

*Family characteristics* included current choice of partnership and whether the subject had children. This was broken into five categories (never married and no partner, never married but currently cohabiting with boyfriend/girlfriend, never married but currently with a non-cohabiting boyfriend/girlfriend, currently married with a child or children, and currently married with no children). Analyses also included two early-adolescence family measures. The first was parental *guan*, usually translated as “parental control”, which involves whether parents allow their adolescent child to decide who his/her friends are when the adolescent was 15 years old. This was coded as either “yes” or “no” [26,27]. Another was family cohesion, which represents the cultural and normative environment for adolescents in Taiwan. It has been hypothesized that this influences their psychological well-being [10,19,20]. The family cohesion scale consisted of four statements: ‘In our family, we have a discussion when making decisions’; ‘Every family member participates in family-related activities’; ‘I can always receive comfort from my family when I feel frustrated’; and ‘I can rely on my family when I need help or advice’. Each item was rated on a four-point scale, indicating the degree of agreement for each cohesive family behavior. A higher score represents stronger cohesion; this measure showed good reliability (Cronbach alpha = 0.82). This scale has been used for prior studies and has been demonstrated to be valid and reliable [20,25].

### Analytical strategy

We assumed that some indicators of loneliness are interrelated, forming loneliness domains. We proposed that there are other indicators that compete with one another, and thus are less likely to cluster in loneliness domains. We conducted latent class cluster analysis (LCA) to assess the underlying structure of loneliness using an exploratory model. This enabled us to determine empirically whether a typology existed regarding the clustering of emotional and social loneliness among all 2,748 young adults [12,13]. LCA is related to more common forms of cluster analysis, but has the added advantages of assigning individuals to classes based on probabilities estimated from the model, as well as being able to provide model fit statistics for choosing between models. A key assumption is that the classes estimated from the models have local independence; that is, independence occurs within each class, while the indicators are assumed to be independent of each other. This means that the latent variable is able to explain why the observed indicators are related to one another [28]. The task of determining the best-fitting 3-class solution is shown in S2 Appendix.

As the next step, we first conducted a 2-part model analysis to assess how these clusters were associated with psychological well-being among all 2,748 young adults. We also assessed how they relate to family characteristics during early adolescence among the 1,609 young adults who had a partner. We first employed multinomial regression techniques to assess the likelihood of being within a certain cluster with regard to psychological well-being (depressive symptoms, self-esteem, drinking behaviors, and suicidal thoughts), partnership choices, and childbearing choices among the 2,748 young adults. As a second step, multinomial regression models were used to assess the associations between family characteristics during early adolescence and the likelihood of being in a certain cluster for the sample of 1,609 young adults who had a partner. In this second part of the analysis, we explored a question that had never previously been asked: do young adults with a cohabiting partner benefit more from having strict parental *guan* during early adolescence? All models considered sample clustering in the survey design, and were analyzed using STATA 15.0 [29].

## Results

Table 1 presents descriptive statistics for the study sample. Ages within the sample ranged from 27 to 32 years old, and 47% were female, while 53% were male. Less than half (47%) of the sample currently lived with their siblings. 41% of the sample reported being single without a partner, while 33% reported having a non-cohabiting partner, 20% reported being married, and 6% indicated they were cohabiting. The mean score for depressive symptoms was 12.31 with a standard deviation of 4.37 and a range between 8 and 37. About one-fifth (24%) abstained from alcohol consumption, while 8% reported binge drinking during young adulthood. Among the young adults investigated in this study, only 3% reported ever having had suicidal thoughts. Overall, 32% of the participants had had a first dating experience by the age of 18.

**Table 1:** Descriptive characteristics of the sample of young adults [percent or mean (SD)].

Variable		Percent or mean (SD)
Age (range: 27–32)		28.32 (1.14)
Female (%)		46.72
Currently employed (%)		88.86
Currently living with siblings (%)		47.23
*Family characteristics*		
Partnership and childbearing choice (%)		
	Single, without a partner	40.98
	Has a partner, cohabiting	6.05
	Has a partner, not cohabiting	33.05
	Married, with children	14.27
	Married, without children	5.65
Family cohesion (range: 1–4)		2.81 (0.65)
Parental *guan* (parental control; %)		34.97
*Psychological well-being*		
Depressive symptoms (range: 8–37)		12.31 (4.37)
Self-esteem (range: 11–36)		26.08 (3.95)
Age at first alcohol use (%)		
	Abstainer	23.69
	Adolescent drinking (17 years old or younger)	25.07
	Adult drinking (18 years old or older)	51.24
Adult binge drinking (%)		8.48
Suicidal thoughts (%)		2.80
Began dating at age 18 or younger (%)		32.06
*N*		2,748

*Note*: SD = standard deviation

**Fig 2:**
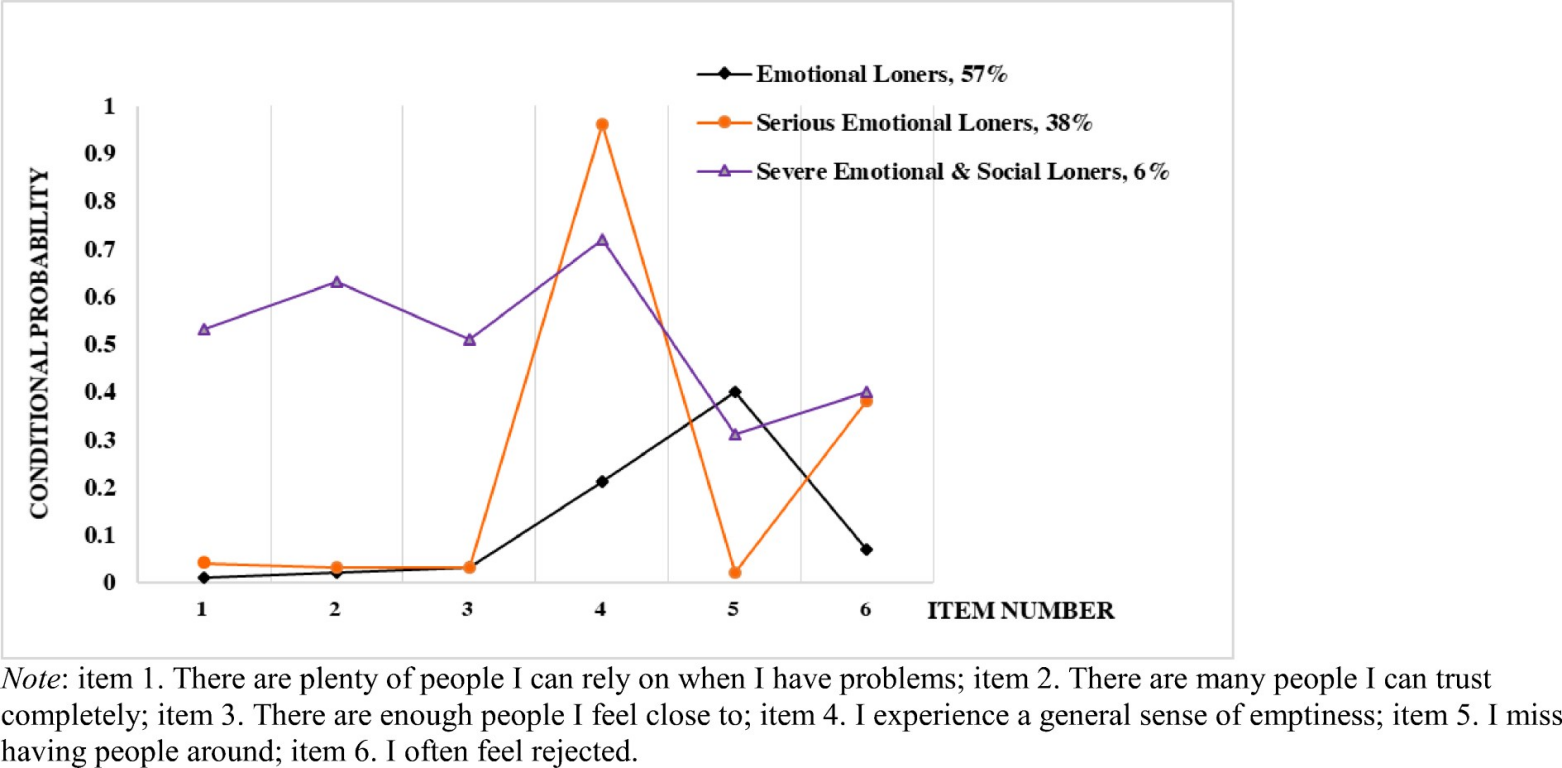
Latent class analysis for conditional probabilities by each loneliness item, stratified by three clusters.

Table 2 shows the conditional probabilities for six items, within three specific clusters. The first loneliness cluster, which we labeled *emotional loners*, contained 57% of the subjects. An inspection of the conditional probabilities indicated that persons in this cluster were likely to report a moderate degree of missing having people around (40%). The second loneliness cluster, *serious emotional loners*, contained 38% of the respondents. Young adults in this cluster showed a modest likelihood of experiencing a general sense of emptiness (96%) and often feeling rejected (38%). The third cluster, labeled *severe emotional and social loners* consisted of 6% of the subjects. These persons demonstrated a moderate likelihood of experiencing all six items, including the emotional and social domains of loneliness. The results from Table 2 are expressed as a graph (see Fig 2).

**Table 2:** Latent class cluster models for conditional probabilities for the three-cluster model of loneliness among young adults.

	Latent Class		
Indicator of Loneliness Item	Emotional Loners	Serious Emotional Loners	Severe Emotional & Social Loners
1. There are plenty of people I can rely on when I have problems.	0.01	0.04	0.53
2. There are many people I can trust completely.	0.02	0.03	0.63
3. There are enough people I feel close to.	0.03	0.03	0.51
4. I experience a general sense of emptiness.	0.21	0.96	0.72
5. I miss having people around.	0.40	0.02	0.31
6. I often feel rejected.	0.07	0.38	0.40
Latent class probabilities	0.57	0.38	0.06

### Loneliness and psychological well-being

To further investigate the latent structure of loneliness, we present the results of a multinomial logistic regression model using the total sample (see Table 3). This demonstrates that psychological well-being, partnership choice and childbearing choice were associated with the likelihood of being in a particular cluster (emotional loners serving as the reference group) when considering age and gender. Statistical significance was set at a *p*-value lower than 0.10 due to the small sample size of the severe emotional and social loner groups (n = 123). In this article, we discuss only statistically significant effects. As compared to the reference group, being a serious emotional loner was associated with increased odds of both depressive symptoms (RRR = 1.14, 95% CI 1.12–1.17) and adult binge drinking (RRR = 1.44, 95% CI 1.04–2.00); it was associated with decreased odds of self-esteem (RRR = 0.82, 95% CI 0.80–0.84). Young adults with suicidal thoughts had higher odds of being severe emotional/social loners than being emotional loners (RRR = 3.02, 95% CI 1.21–7.56). Being a severe emotional/social loner was associated with increased odds of depressive symptoms (RRR = 1.15, 95% CI 1.10–1.20) and decreased odds of self-esteem (RRR = 0.78, 95% CI 0.73–0.83), as compared to the reference group.

**Table 3:** Multivariate multinomial logistic regression results for latent structure of loneliness among young adults, N = 2,748.

		Loneliness Cluster/Class Contrast	
Covariate		Serious Emotional Loners *vs*. Emotional Loners RRR (95% CI)	Severe Emotional & Social Loners *vs*. Emotional Loners RRR (95% CI)
**Psychological well-being**			
Depressive symptoms		1.14 (1.12, 1.17)**	1.15 (1.10, 1.20)**
Self-esteem		0.82 (0.80, 0.84)**	0.78 (0.73, 0.83)**
Suicidal thoughts (ref = no)		1.31 (0.56, 3.05)	3.02 (1.21, 7.56)*
Age at first alcohol use (ref = adult drinking)			
	Abstainer	0.85 (0.69, 1.05)	1.56 (1.00, 2.44)^§^
	Adolescent drinking	0.79 (0.63, 0.98)*	0.75 (0.44, 1.29)
Adult binge drinking (ref = no)		1.44 (1.04, 2.00)*	0.81 (0.34, 1.95)
**Individual/family characteristics**			
Partnership and childbearing choice (ref = Has a partner, not cohabiting)			
	Single, without a partner	2.11 (1.73, 2.58)**	1.89 (1.22, 2.93)**
	Has, a partner, cohabiting	0.74 (0.51, 1.07)	1.14 (0.55, 2.37)
	Married, with children	0.79 (0.58, 1.08)	0.54 (0.26, 1.13)
	Married, without children	0.64 (0.44, 0.93)*	0.96 (0.40, 2.30)
Age		0.95 (0.87, 1.03)	1.11 (0.94, 1.31)
Female		0.87 (0.70, 1.09)	0.74 (0.49, 1.12)

Abbreviations: RRR = relative risk ratio; CI = confidence interval.

Note

^§^p < 0.10

*p < 0.05

**p < 0.01.

In addition, adolescent drinkers were less likely than adult drinkers to be in the serious emotional loners cluster (RRR = 0.79, 95% CI 0.64–0.98), whereas abstainers were more likely than adult drinkers to be in the severe emotional/social loners cluster (RRR = 1.56, 95% CI 1.00–2.44). Net of the other variables in the model, single young adults were more likely than those young adults who had a non-cohabiting partner to be serious emotional loners (RRR = 2.11, 95% CI 1.73–2.58) or severe emotional/social loners (RRR = 1.89, 95% CI 1.22–2.93), as compared to the reference cluster (emotional loners). Childless married young adults were less likely than young adults with a non-cohabiting partner to be in the serious emotional loners cluster, as compared to the reference cluster (RRR = 0.64, 95% CI 0.44–0.93).

### Loneliness and family characteristics among partnered young adults

The establishment of a stable intimate relationship is a major transition of young adulthood, thus we focus on the partnered sample. Table 4 shows the results of the multinomial logistic regression models analyzed by loneliness clustering among partnered young adults. Building on the risky families model, we included family context variables measuring partnership choice, childbearing choice, family cohesion, parental *guan* and age when dating began, while adjusting for a wide range of individual covariates such as gender, age, and current work status. This model investigated the association between the four categories of explanatory partner variables and the odds of being serious emotional loners and severe emotional/social loners, compared to being emotional loners. Married young adults with at least one child were less likely than young adults who had a non-cohabiting partner, to report loneliness, either serious emotional loneliness (RRR = 0.74; 95% CI 0.55–1.01) or severe emotional/social loneliness (RRR = 0.51; 95% CI 0.25–1.03). The odds of loneliness, both emotional and social, decreased as adolescent family cohesion increased, while the odds of serious emotional loneliness increased as adolescent parental *guan* increased. In addition, a significant effect of cohabitation involving an interaction with adolescent parental *guan* was observed (RRR = 0.54, 95% CI 0.27–1.09); this indicates that young adults who reported strict parental *guan* during early adolescence have higher odds of reporting serious emotional loneliness in general (RRR = 1.27, 95% CI 1.02–1.59). On the other hand, young adults, who cohabit, and who also report strict adolescent parental *guan*, had lower odds of being serious emotional loners. Young adults who had first dated at the age of 18 or earlier were more likely to report severe emotional/social loneliness than their counterparts who had started dating later.

**Table 4:** Multivariate multinomial logistic regression results for family characteristics associated with latent structure of loneliness in young people with a partner, N = 1,609.

		Loneliness Cluster/Class Contrast	
Covariate		Serious Emotional Loners *vs*. Emotional Loners RRR (95% CI)	Severe Emotional & Social Loners *vs*. Emotional Loners RRR (95% CI)
Choices of partnership and childbearing (ref = Has a partner, not cohabiting)			
	Has a partner, cohabiting	1.10 (0.73, 1.65)	1.61 (0.73, 3.57)
	Married, with children	0.74 (0.55, 1.01)^§^	0.51 (0.25, 1.03)^§^
	Married, without children	0.64 (0.44, 0.93)*	0.99 (0.42, 2.31)
First dating at age 18 or younger		1.08 (0.86, 1.35)	1.69 (1.01, 2.83)*
*Family characteristics in early adolescence (at aged 15)*			
Family cohesion		0.77 (0.65, 0.91)**	0.54 (0.34, 0.85)**
Parental guan		1.27 (1.02, 1.59)*	0.57 (0.29, 1.12)
*Comparison between cohabiting and non-cohabiting young people*			
	Cohabiting partner × Guan	0.54 (0.27, 1.09)^§^	0.61 (0.12, 2.99)
	*Individual characteristics*		
	Currently employed (ref = No)	0.73 (0.53, 1.01)^§^	0.82 (0.36, 1.91)
	Female	1.36 (1.08, 1.72)*	1.25 (0.72, 2.16)
	Age	0.98 (0.89, 1.09)	1.32 (1.06, 1.65)*

Abbreviations: RRR = relative risk ratio; CI = confidence interval.

Note

^§^p < 0.10

*p < 0.05

**p < 0.01.

In addition, serious emotional loneliness was found to be associated with decreased odds of being employed (RRR = 0.73, 95% CI 0.53–1.01) and increased odds of being female (RRR = 1.36; 95% CI 1.08–1.72). Finally, the odds of being a severe emotional and social loner increased as young adults aged (RRR = 1.32, 95% CI 1.06–1.65).

## Discussion

The purpose of this study was to explore loneliness clusters using a large community sample of young persons. We examined whether domains of loneliness, or loneliness clusters, form during young adulthood. Are these clusters associated with psychological well-being? Are choices regarding partnership and childbearing, as well as family context during early adolescence, associated with specific loneliness clusters in early adulthood? To explore these questions, we assumed that some indicators of loneliness are interlinked, forming loneliness domains. Furthermore, we proposed that other indicators are in competition with one another, and are thus less likely to cluster into loneliness domains. To explore these possibilities, we examined data from the TYP survey and employed LCA to identify various latent classes of loneliness. We also used multinomial logistic regression techniques to investigate how the identified clusters were associated with psychological well-being and family characteristics.

Our results show the existence of a latent structure within loneliness. LCA showed that a three-cluster model captured the underlying relationships between the social and emotional loneliness indicators; the identified clusters consisted of emotional loners, serious emotional loners, and severe emotional/social loners. The model indicated that the emotional and social domains of the loneliness indicators are complementary, at least for some of the study sample (severe emotional/social loners). This result is consistent with the literature [7]. Young adults in this cluster combine emotional and social isolation, which implies that these are complementary indicators of the loneliness concept. In addition, experiencing a general sense of emptiness and missing having people around, two of the discretionary indicators, are common. Interestingly, a small group of young adults seemed to experience severe emotional and social loneliness. These persons mix the emotional and social indicators of loneliness, and in doing so have greater risk of depressive symptomatology and suicidal thoughts. Further investigation of this group may find that they suffer from an unusually large number of mental health problems and/or more severe mental health problems later in life. This may also be correlated with family adversity during early adolescence.

Young and partnered people with strong family cohesion during early adolescence were less likely to be serious emotional loners or severe emotional/social loners, regardless of gender. This is consistent with the hypothesis of the *risky families model* [10] in family conflict and mental health. Serious emotional loners appear to be more common among partnered young adults who reported stricter parental *guan* during early adolescence, although this association was found to level off among cohabiting adults. Our results suggest a risky family environment early in life, and negative consequences of mental health in later life. Taiwan, similar to other East Asian countries such as Japan and South Korea, has a historical background in Confucian ideology. This underscores the popular Chinese slogan “*jia he wan shi xing*” (harmony in the family is the basis for success in any undertaking). This common *tradition* appears to be a fundamental force that even shapes mental health, such as young adults’ loneliness in this cultural context.

Both personal and cultural background may guide researchers’ understanding of loneliness, the experience of which varies among young adults. Our analysis indicates that loneliness clusters are related to various dimensions of psychological well-being including depressive symptoms, self-esteem, suicidal thoughts, and alcohol consumption [4,30,31]. This is similar to how this study has shown how partnership and childbearing choices, as well as family context during early adolescence, are related to loneliness clusters. Several research questions arise from these findings: To what degree are loneliness clusters associated with worse (psychological and physical) well-being, from a life-course perspective? Are the factors associated with being in one loneliness cluster versus another the same for different socioeconomic groups and/or social networks? To what degree are clusters of loneliness shaped in a family context by choices regarding partnership and childbearing? In addressing the above questions, researchers should account for the endogeneity and reciprocal causation that may exist between the loneliness clusters and well-being. Researchers may also need to consider the interrelationship between socioeconomic status and family context.

There are several limitations to this study. First, loneliness was measured using a six-item scale that is subject to recall bias. In addition, young adults were asked to respond to questions about their loneliness, and identifying with a status may have had a stigma attached. Rather than a single-item question, however, we adopted a validated six-item scale [12,13,32,33]. The second problem is endogeneity. There is a simultaneous causation between choices regarding partnership, childbearing and the loneliness clusters. In such circumstances, a cross-sectional study approach is likely insufficient for disentangling this link. Thirdly, although the analysis controlled for socio-demographic factors, we were unable to control for several important individual characteristics such as age, gender, and current work status; nor were we able to control for family dynamics and friendship influences. Friendship influences may be associated with factors within social networks, and these are also associated with loneliness [34]. Fourth, in a panel sample, attrition is always a concern. Although the group of participants with missing data was more likely to be unhealthy, maximizing sample size and retaining sample diversity remain important considerations.

To our knowledge, this is the first report of a latent structure of loneliness among young adults in a non-Western society. Three clusters of loneliness are associated with other indicators of psychological well-being. Family environment in general, and strong family cohesion during early adolescence in particular, appear to protect from becoming a serious emotional loner or a severe emotional/social loner during young adulthood, in a non-Western society. Future research is needed to better understand loneliness clustering in a broader relationship context. This will enable policy makers to utilize this information to develop more effective prevention programs and organize interventions that alleviate young adult loneliness in multiple domains [2] and even other demographic groups, such as older adults [1,35].

## Supporting information

S1 Appendix 6-item de Jong-Gierveld short scale and its descriptive statistics and psychometrics properties used in TYP, N = 2,748. (DOC)

S2 Appendix Latent class cluster models of loneliness among young adults using the 6-item de Jong-Gierveld short scale. (DOC)
