# Supplementary material for: Loneliness in young adulthood: Its intersecting forms and its association with psychological well-being and family characteristics in Northern Taiwan
Source: PLoS One. 2019 May 31;14(5):e0217777. doi: 10.1371/journal.pone.0217777 (PMC6544274; doi:10.1371/journal.pone.0217777)
Supplement: S1 Appendix — (DOC) [file pone.0217777.s001.doc]

Appendix **1**. 6-item de Jong-Gierveld short scale and its descriptive statistics and psychometrics properties used in TYP, N=2,748

|  | | **Continuous Score^1^** | | | **Dichotomous Score^2^** | | |
| --- | --- | --- | --- | --- | --- | --- | --- |
|  | | Mean (Std Dev) | CFA^3^: factor loading | | Percent | CFA: factor loading | |
| **6-item de Jong-Gierveld short scale** | |  | Emotional | Social |  | Emotional | Social |
| 1. There are plenty of people I can rely on when I have problems. | | 1.41 (0.59) | **0.71** | -0.13 | 5.13 | **0.71** | 0.10 |
| 2. There are many people I can trust completely. | | 1.41 (0.60) | **0.79** | -0.04 | 5.75 | **0.78** | 0.06 |
| 3. There are enough people I feel close to. | | 1.40 (0.59) | **0.67** | -0.07 | 5.64 | **0.69** | 0.11 |
| 4. I experience a general sense of emptiness. | | 0.58 (0.61) | -0.19 | **0.67** | 52.00 | 0.16 | **0.64** |
| 5. I miss having people around. | | 0.99 (0.71) | 0.08 | **0.53** | 25.33 | 0.03 | **0.49** |
| 6. I often feel rejected. | | 0.22 (0.45) | -0.19 | **0.37** | 20.71 | 0.17 | **0.37** |
|  | |  |  |  |  |  |  |
| Model fit | |  |  |  |  |  |  |
|  | CFI | 0.96 |  |  | 0.99 |  |  |
|  | TLI | 0.93 |  |  | 0.98 |  |  |
|  | SRMR | 0.05 |  |  | 0.03 |  |  |
|  | Correlation between latent factors | 0.30 |  |  | 0.34 |  |  |
|  | Cronbach’s α | 0.62 | 0.50 | 0.77 | 0.66 | 0.50 | 0.70 |

*Note*: ^1^ Average individual current experiences score for each item, ranging from no (0), more or less (1), to yes (2)

^2^ Each item was recoded into a dichotomous score, indicating percent who felt intensely lonely [13]

^3^ CFA: confirmatory factor analysis after varimax rotation
