# Supplementary material for: Loneliness in young adulthood: Its intersecting forms and its association with psychological well-being and family characteristics in Northern Taiwan
Source: PLoS One. 2019 May 31;14(5):e0217777. doi: 10.1371/journal.pone.0217777 (PMC6544274; doi:10.1371/journal.pone.0217777)
Supplement: S2 Appendix — (DOC) [file pone.0217777.s002.doc]

Appendix **2**. Latent class cluster models of loneliness among young adults using the 6-item de Jong-Gierveld short scale

| Model | L^2^ | *df* | *p* | BIC | AIC |
| --- | --- | --- | --- | --- | --- |
| One-cluster model | 970.71 | 57 | 0.00 | 13279.79 | 13244.28 |
| Two-cluster model | 396.38 | 50 | 0.00 | 12760.90 | 12683.95 |
| Three-cluster model | 80.93 | 43 | 0.00 | 12500.87 | 12382.50 |

*Note*: AIC = Akaike's information criterion; BIC = Bayesian information criterion
